# Implication of thermally conductive nanodiamond-interspersed graphite nanoplatelet hybrids in thermoset composites with superior thermal management capability

**DOI:** 10.1038/s41598-019-39127-z

**Published:** 2019-02-27

**Authors:** Yinhang Zhang, Mira Park, Soo-Jin Park

**Affiliations:** 1 Department of Chemistry, Inha University, 100 Inharo, Incheon, 22212 Korea; 2 Department of Bioenvironmental Chemistry, College of Agriculture & Life Science, Chonbuk National University, Jeonju, 54896 South Korea

## Abstract

Novel hybrid nanofillers composed of nanodiamond-attached graphite nanoplatelets (ND@GNPs) were designed and employed to toughen the epoxy (EP) matrix for fabricating superior thermal conductive and physically robust thermoset nanocomposites for electronics and auto industries. The hybrid nanofiller was covalently bonded by 4,4′-diphenylmethane diisocyanate and it provided distinct enhancement in thermal conductivity and dynamic storage modulus of the EP/ND@GNPs nanocomposites attributing to the unique nanostructure of ND@GNPs that can form strong interfacial interaction with EP matrix, thus restrict the EP molecular motions. The EP/ND@GNPs20 presented a thermal conductivity of 2.48 W · m^−1^ · K^−1^ and dynamic storage modulus of 5.6 GPa. The presence of ND particles not only can enhance heat transfer efficiency but also improve the interfacial interaction between ND and EP matrix, which can directly affect physical properties of the EP composites.

## Introduction

The continuous miniaturization and increasing power density of thermal-management-related electronic devices, such as CMOS, LEDs, automotive, and aerospace products, means that thermally conductive and physically strong materials are becoming increasingly important in tandem with the need for more efficient heat diffusion and easy processing^1–6^. Polymer-based materials possess excellent dielectric properties and are readily processed, but have poor thermal conductivity (mostly below 0.2 W · mK^−1^)^7–10^. Consequently, polymer composites reinforced using particles with high thermal conductivity have the potential to be ideal electronic packaging materials capitalizing on the particles’ high thermal conductivity and the ease of polymer processing. Generally, the reinforced polymer composites also possess robust thermal-physical properties.

Recent studies have shown that by incorporating thermally conductive particles, such as boron nitride (BN)^11–15^, silicon carbide^16–18^, aluminum oxide^19,20^, and various carbon-based fillers^21–26^, the thermal conductivity of reinforced polymer composites can be significantly enhanced. However, among the fillers, ceramic-based particles cannot effectively enhance the thermal conductivity of polymer composites. In Wu *et al*.’s study^27^, styrene-butadiene rubber (SBR) and sulfide-containing silane-modified BN composites were prepared by compounding the BN slurry with SBR via conventional two-roll milling. Even with *in*-*situ* modification of silane, it was found that the thermal conductivity of the composites with 10.5 vol% BN could only reach 0.57 W · m^−1^ K^−1^. Yu *et al*.^28^ investigated the thermal conductivity of a polymer composed of a matrix of polystyrene containing aluminum nitride (AlN) particles. They found that the thermal conductivity reached approximately 1 W · m^−1^ K^−1^ with an AlN volume fraction of 40%. Wu *et al*. used an *in*-*situ* synthesis method to introduce zirconium diboride (ZrB_2_) particles with high thermal conductivity into an epoxy polymer matrix to improve the thermal conductivity of the epoxy matrix. They found that the thermal conductivity of the composites was only 0.36 W · m^−1^ K^−1^ at 25 °C with the ZrB_2_ content of 16% mass fraction. Compared with the ceramic particle-incorporated polymer composites, the carbon-based particle fillers generally resulted in higher thermal conductivity with a lower mass fraction or volume fraction in the polymer matrix. Min *et al*.^24^ developed graphite nanoplatelet (GNP)/epoxy composites through a typical interface design; these composites had a thermal conductivity of 0.72 W · m^−1^ K^−1^ with a very low loading of 2.7 vol%. Song *et al*.^2^ fabricated epoxy/graphene nanocomposite flakes by dispersing non-oxidized graphene flakes with a non-covalent functionalization of 1-pyrenebutyric acid in an epoxy matrix. With 10 wt% graphene flakes, the thermal conductivity of the material reached 1.53 W · m^−1^ K^−1^ and the mechanical properties were enhanced. In Lian *et al*.’s study^29^, a changeable work had been down by incorporating vertically aligned and interconnected graphene networks in the epoxy matrix. The resulting composite, with an ultralow graphene loading of 0.92 vol%, presented a significantly high thermal conductivity of 2.13 W · m^−1^ K^−1^; this is equivalent to an enhancement of 1231% compared to the pure matrix.

Beyond the intrinsic properties of fillers, the most widely used methods for enhancing the thermal conductivity of filler-reinforced composites are surface functionalization of fillers^30,31^, constructing core-shell additives^32^, and incorporating hybrids and utilizing the synergistic effect of the fillers^33–35^. To take advantage of all three of these methods in this work, nanodiamond (ND) nanocluster attached and supported graphite nanoplatelet (GNP) hybrids were prepared and utilized to construct a thermally conductive network in an epoxy matrix. The constructed 3D nanostructure provides a path of lower resistance for phonons, considerably enhancing the thermal conductivity of the nanocomposites.

## Results and Discussion

**Figure 1:**
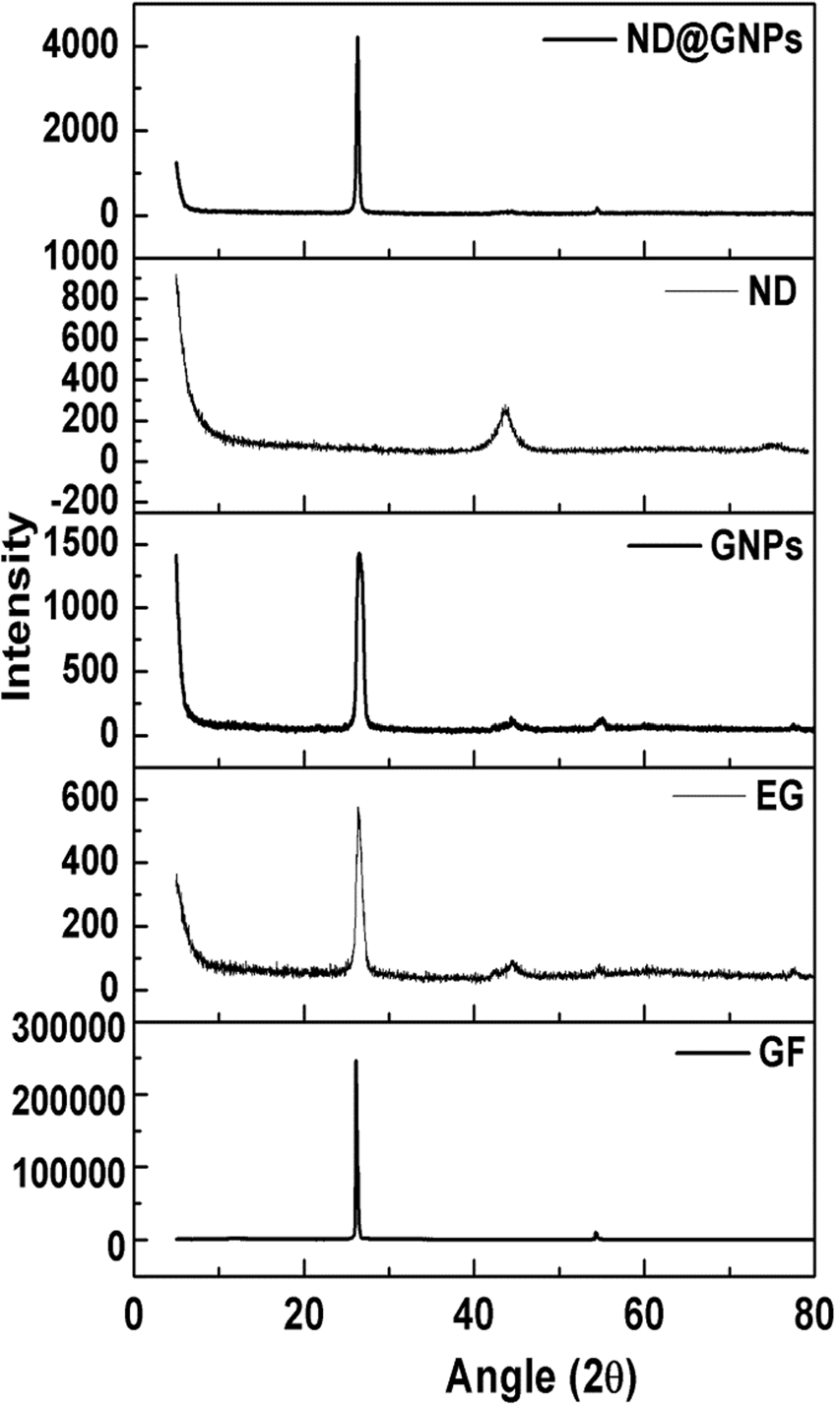
XRD patterns of the particles.

XRD was conducted to exam the structure of the various fillers and the XRD patterns are shown in Fig. 1. The characteristic peak of the GFs appears at 2θ = 26.2° and corresponds to the (002) interlayer. After expansion, the typical (002) peak of expanded graphite broadens and weakens compared to pristine GFs. The ND diffraction pattern gives two characteristic peaks at 2θ = 43.7 and 75.5°, which correspond to the (111) and (220) diamond planes respectively, demonstrating that the grown ND crystals are cubic^36^.

**Figure 2:**
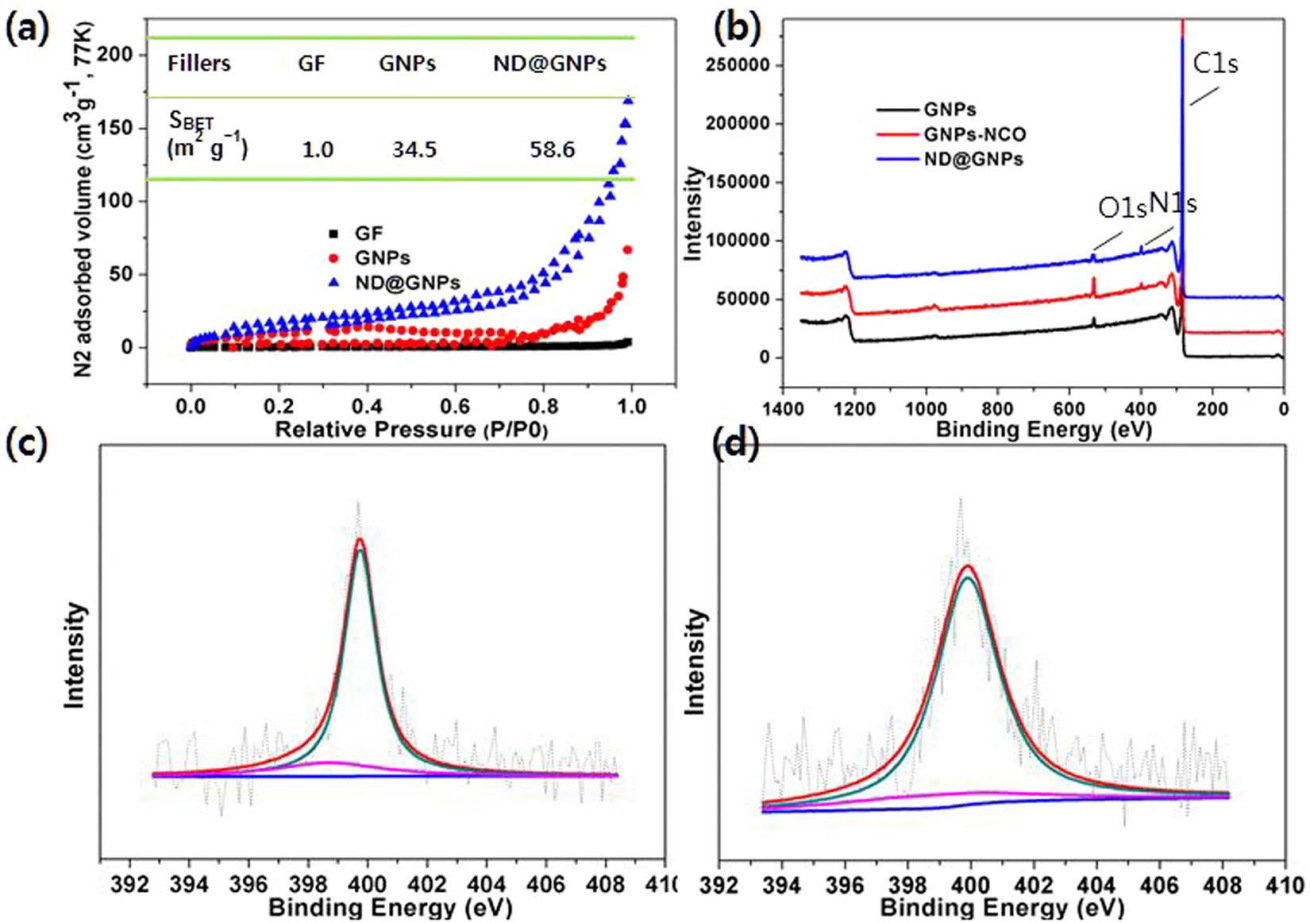
(**a**) N_2_ adsorption/desorption isotherms of GF, GNPs and ND@GNPs; (**b**) XPS survey of the GNPs, GNPs-NCO and ND@GNPs; High-resolution XPS spectra of N1s of (**c**) GNPs-NCO and (**d**) ND@GNPs.

It is well known that specific surface area is an important structural characteristic of the filler in composite systems; high values represent a higher interfacial interaction per unit volume, which can affect the chemical and physical properties of the composites^37^. Nitrogen adsorption was employed to estimate the specific surface area of the GFs, GNPs, and ND@GNPs; the sorption isotherms are presented in Fig. 2(a). As expected, the pristine GFs gave a much lower specific surface area of 1.01 m^2^ · g^−1^ due to their large particle size. After expansion and exfoliation, the GNPs showed a higher specific surface area of 34.5 m^2^ · g^−1^. The ND@GNPs had a value of 58.6 m^2^ · g^−1^, attesting to the high specific surface area of the interspersed nanodiamond nanoclusters^7,38^.

XPS survey spectra were collected to confirm the surface chemical compositions of the GNPs, GNPs-NCO, and the ND@GNPs. Strong C1s peaks were detected for all of the samples due to the high carbon content. N1s peaks were observed in both the GNPs-NCO and ND@GNPs due to the covalently attached –NCO groups in the MDI modules, demonstrating the successful grafting of MDI. Figure 2(c and d) show the high-resolution single scan spectra of the N1s region for the GNPs-NCO and ND@GNPs, respectively. The GNPs-NCO presented two peaks at 398.8 and 399.7 eV, corresponding to the C=N–C and N–COO groups, respectively. After ND attachment, the C=N–C groups were consumed to react with the –OH on the ND surfaces, resulting in the almost complete disappearance of the C=N–C peak at 398.7 eV in the ND@GNPs.

**Figure 3:**
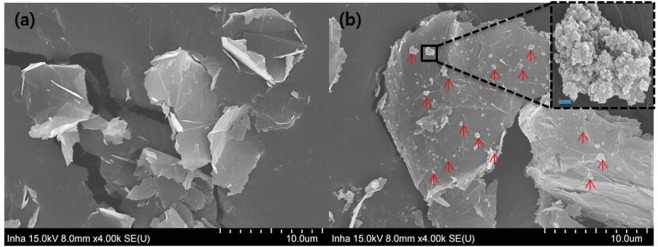
Surface morphology of the GNPs and ND@GNPs.

The SEM morphologies of GNPs and ND@GNPs are shown in Fig. 3(a and b), respectively. The GNPs are seen to be irregularly shaped and visual inspection gives a particle size in the range of 0–10 μm and a thickness of less than 100 nm. In the case of the ND@GNPs, nanodiamond nanoclusters with particles less than 1 μm in diameter were interspersed on the GNP surfaces. No separated nanodiamond nanoclusters were detected on the conductive adhesive, representing a strong binding force between the nanodiamond nanoclusters and the GNPs.

**Figure 4:**
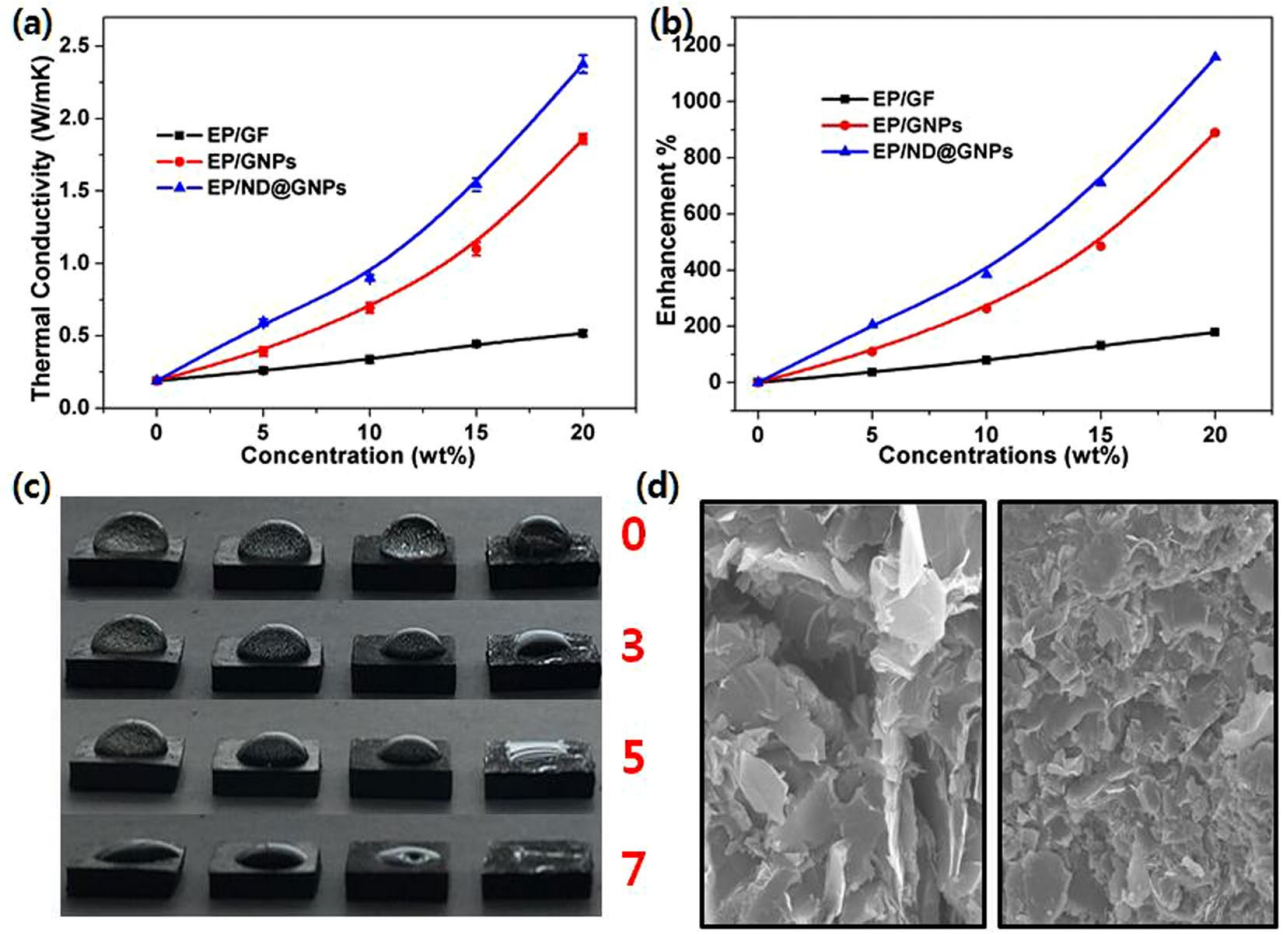
(**a**) Thermal conductivity and (**b**) percentage enhancement of the conductivities of EP/GF, EP/GNPs and EP/ND@GNPs composites as a function of filler concentration; (**c**) the digital photograph image of water evaporation above EP (left), EP/GF20 (middle left), EP/GNPs20 (middle right) and EP/ND@GNPs20 (right) with time (min), and (**d**) SEM fractograph of EP/GNPs20 (left) and EP/ND@GNPs20 (right).

Figure 4(a) shows the thermal conductivities of the EP/GF, EP/GNPs, and EP/ND@GNPs as a function of related filler concentration, where the EP/GF and EP/GNPs are provided for comparison. It can be seen that the ND@GNPs structure resulted in a dramatic enhancement of the thermal conductivity of the EP/ND@GNPs composites compared to the pure epoxy resin, which has thermal conductivity of only 0.19 W · m^−1^ K^−1^ due to its amorphous nature. With a filler concentration of 20 wt%, the EP/ND@GNPs presented a thermal conductivity of 2.48 W · m^−1^ K^−1^, which is an enhancement of 1205% compared to the neat EP (Fig. 4(b)). At the same concentration, the EP/GF20 and EP/GNPs20 exhibited a 168% and 894% enhancement of thermal conductivity compared to the neat EP, respectively. It is interesting to observe that starting with 10 wt% of ND@GNPs enhanced the thermal conductivity of the EP/ND@GNPs nanocomposites faster, indicating the formation of a systematic conductive network.

Figure 4(c) shows the digital images of the water evaporation on the surfaces of EP, EP/GF20, EP/GNPs20, and EP/ND@GNPs20. Since a difference in surface free energy can affect the contact angle and contact area of water drops on the sample surface, all the samples were treated with a graphite coating. The temperature of the heating plate was kept at 100 °C and the water evaporated from the sample in the sequence of EP/ND@GNPs20, EP/GNPs20, EP/GF20, and EP. This suggests that, the novel and efficient 3-D structure of the ND@GNPs is responsible for the distinctively enhanced thermal conductivity of the EP/ND@GNPs composites.

**Figure 5:**
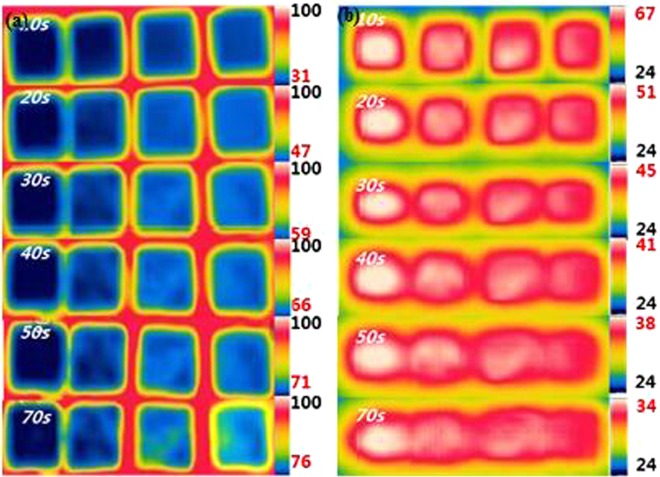
(**a**) Infrared thermal images during heating process from 18 °C; (**b**) infrared thermal images during the cooling process of EP (left), EP/GF20 (middle left), EP/GNPs20 (middle right) and EP/ND@GNPs20 (right).

**Figure 6:**
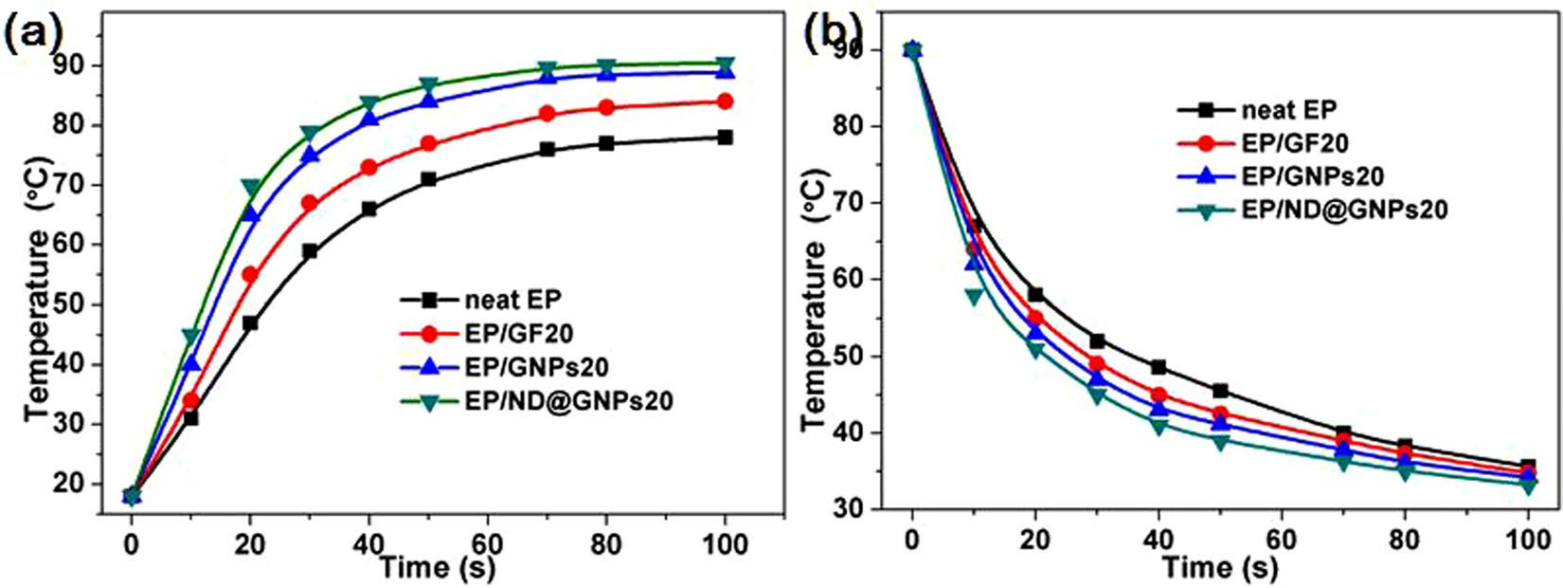
(**a**) Surface temperature variations during the heating process; surface temperature variations during cooling process from 90 °C of EP (left), EP/GF20 (middle left), EP/GNPs20 (middle right) and EP/ND@GNPs20 (right).

The surface temperature variation over time of the EP, EP/GF20, EP/GNPs20, and EP/ND@GNPs20 composites during cooling and heating were recorded with an infrared camera to demonstrate the thermal management applications of the composites. The optical photos and the temperature variation with time are shown in Fig. 5. To investigate the heat absorption capabilities, the above-mentioned samples were placed vertically on a glass plate with a temperature of 110 °C. It is worth noting that the temperature of the EP/ND@GNPs increased the quickest and was close to 90 °C after just 100 s due to its superior thermal conductivity. The other composites heated more slowly and took much longer to reach to the heat plate temperature. In the case of the heat dissipation performance, the samples were kept in the oven at a temperature of 90 °C until all the samples were at a uniform stable temperature. They were then placed on the glass plate simultaneously to ensure the same initial temperature. It can be seen that during the heat dissipation process, the temperatures of the EP/ND@GPN20 composite decreased faster than the others. This is because the EP/ND@GPNs composite has a better thermal response to the external environment due to its superior thermal conductivity. All the results demonstrate that the EP/ND@GNPs nanocomposite has high potential for thermal management applications. The related surface temperature variations during the heating and cooling process are summarized in Fig. 6(a and b), respectively.

**Figure 7:**
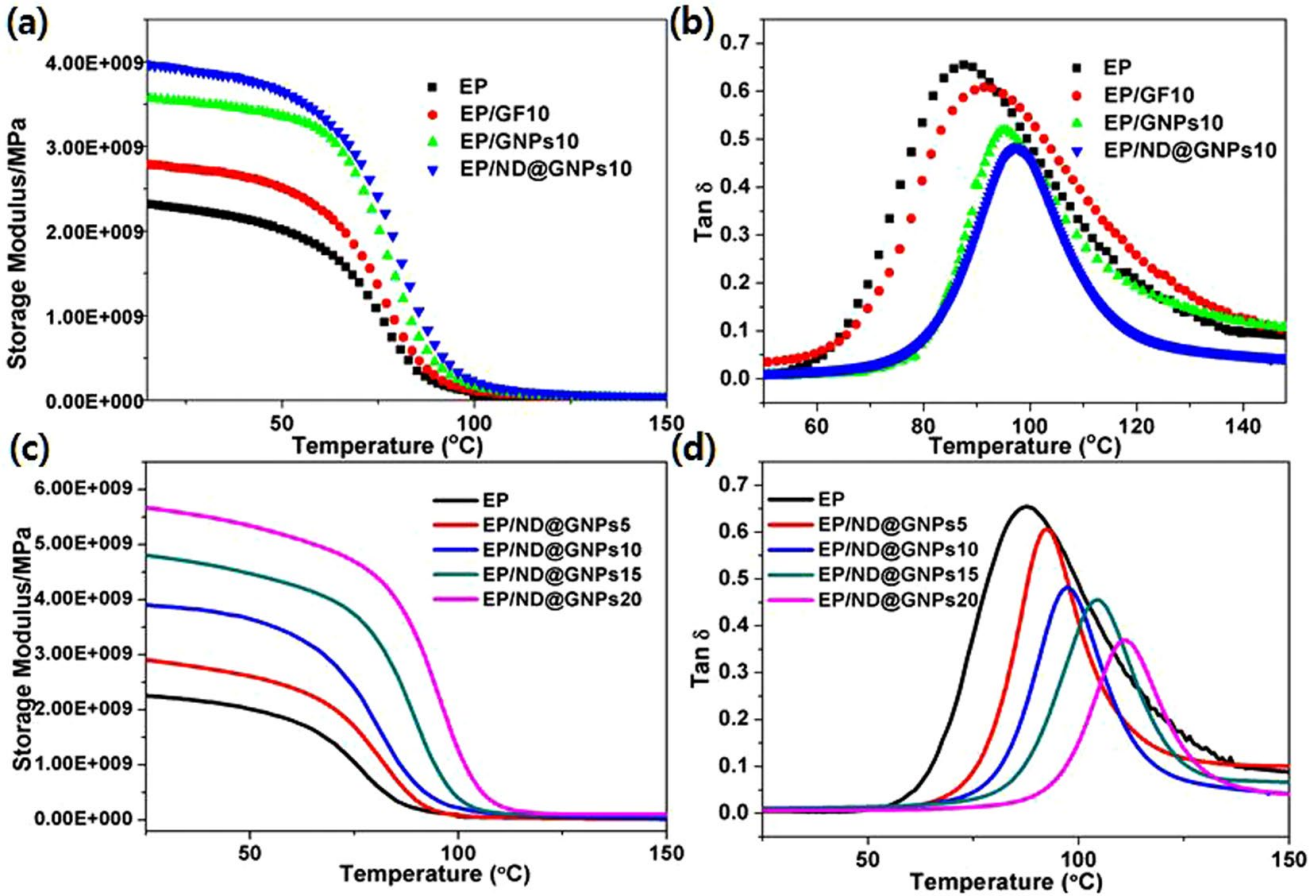
(**a**) Storage modulus and (**b**) damping factor (Tan δ) of EP, EP/GF10, EP/GNPs10 and EP/ND@GNPs10 composites; (**c**) Storage modulus and (**d**) damping factor (Tan δ) of EP and its composites as a function of ND@GNPs concentration.

Dynamic mechanical analysis of the neat epoxy and the epoxy-based composites can provide information about the storage modulus and loss factor (tan δ), defined as the ability of the composites to store elastic energy and dampening ability, respectively. It can also be employed to determine the Tg, where sufficient vibrational energy has accumulated to rearrange the crosslinked polymer chains in the polymer matrix (also known as the relaxation behavior), which is very sensitive to structural transformations^39^. The effect of the filler type on the storage modulus and Tg of the EP, EP/GF10, EP/GNPs10, and EP/ND@GNPs10 composites at a filler concentration of 10 wt% is shown in Fig. 7(a and b), respectively. It can be seen that the storage modulus of all four composites continuously decreases with temperature due to the softening of the epoxy chains with increasing temperature. The EP/ND@GNP composite exhibited the highest initial storage modulus which was attributed to the special structure of the ND@GNPs that can directly restrict the polymer chain mobility. Moreover, the EP/ND@GNP composite showed the highest Tg value, due to the enhanced interfacial interaction and contact area between the filler and the epoxy matrix. The enhanced interfacial interaction could also be proved by the comparison of the fracture surface morphology which is shown in Fig. 4(d).

The temperature dependences of the storage modulus and the loss factor of the neat EP and EP/ND@GNP nanocomposites as a function of filler concentration are shown in the Fig. 7(c and d), respectively. The EP/ND@GNP composites show a significantly higher storage modulus than the neat EP and the storage modulus values at the initial glassy state were enhanced with filler concentration. The improvement in the thermo-physical performance could be ascribed to the unique nanostructure of ND@GNPs, which can effectively restrict the molecular motions and readily transfer the external load from matrix to the rigid inorganic fillers. The Tg values of the EP/ND@GNP nanocomposites increased with filler concentration, meaning that the ND@GNPs rigid nanostructure could hinder the motion of the epoxy chains at high temperatures. The DMA results demonstrated that the ND@GNPs could effectively increase the dynamic mechanical behaviors of the EP-based composites.

## Conclusions

Novel hybrid ND@GNPs was designed for the first time and their covalent binding force was confirmed. ND particles not only increase thermally conductive paths to enhance heat transfer and dissipation, but also increase interfacial interactions to restrict molecular motion to improve the thermo-physical properties of the composites.” ND@GNP-toughened epoxy composites were fabricated. As anticipated, these composites exhibited superior thermal conductivity and thermo-physical properties which was attributed to the unique structure of the ND@GNPs. The EP/ND@GNPs20 presented a thermal conductivity of 2.48 W · m^−1^ · K^−1^, which is an enhancement of 1205% compared to the neat EP. Simultaneously, the composite exhibited a high dynamic storage modulus and Tg value. All the results indicate that the EP/ND@GNP nanocomposites are a promising candidate for thermal management applications in electronics and the automotive industry.

## Methods

### Materials

Natural graphite flakes (GFs, 500 μm), ND, and 4,4′-methylenebis(phenyl isocyanate) (MDI) were purchased from Sigma–Aldrich Co., Korea. The epoxy (YD-128), with an epoxide equivalent weight of 185–190 g/eq at room temperature, was purchased from Kukdo Chemical Co., Korea. The hardener 4,4′-diaminodiphenylmethane (DDM), was supplied by TCI Co., Japan. Sulfuric acid (98%) was supplied by Daejung Chemicals, Co., Korea.

### Synthesis of EG, GNPs, GNPs-NCO, and ND@GNPs

The GFs were charged into sulfuric acid at 70 °C for 1 day. The solution was filtered and washed with distilled water and then dried in a vacuum oven at 80 °C for 24 h. The product was then treated in a muffle furnace at 1000 °C for 120 s and the EG was obtained. GNPs were obtained by treating the EG in acetone and sonicating it for 8 h. To synthesize the MGNPs, the GNPs were charged in MDI-DMF solutions. The reaction was carried out at 80 °C for 24 h, and the solution was then washed with dichloromethane to remove the excess MDI, the solid product was designated as MGNPs.

ND and MGNP powders were dispersed in a DMF solution and exfoliated by ultrasonication for 10 min. The two solutions were then mixed in a round-bottom 500 mL flask with a Teflon-coated magnetic stirring bar, and the reflux was heated to 80 °C for 12 h. After cooling to room temperature, the sediment (ND@GNPs) was collected by filtration, washed with deionized water, and dried in the freeze-dryer for 48 h.

### Preparation of EP/GF, EP/GNP, and EP/ND@GNP composites

A latex compounding technique was employed to fabricate the composites. The desired quantity of filler was dispersed in acetone by sonication for 30 min, followed by charging epoxy resin at 100 °C with vigorous stirring until the solvent was absolutely removed. The hardener, DDM (17 wt%), was dropped into the mixture under mechanical mixing at 150 rpm for 20 min, and was then degassed at 70 °C for 20 min. The epoxy composites were finally obtained after the following curing process: 1 h at 110 °C, 2 h at 140 °C, and 1 h at 170 °C.

### Characterization

X-ray diffraction (XRD, D2 Phaser, Bruker) was performed at room temperature from 4° to 80° (2θ) at a rate of 5° · min^−1^. N_2_ adsorption-desorption isotherms were carried out at 77 K using a gas adsorption analyzer (BEL–SORP; BEL Co., Japan). X-ray photoelectron spectroscopy (XPS, VG Scientific Co., ESCA LAB MK-II) analyses were employed to detect the surface chemistry of the various fillers. The morphology and structure of the fracture surfaces of the composites were investigated using high-resolution scanning electron microscopy (HR-SEM; SU8010, Hitachi Co., Ltd). Dynamic mechanical thermal analysis was conducted using rectangular test specimens with dimensions of 25 mm × 5 mm × 1 mm from 10–150 °C with a dual cantilever clamp and a dynamic mechanical analyzer (DMA Q800, TA instruments). For each kind of composites, three samples were tested.

The thermal conductivity of the composites was determined using an LFA 447 Nanoflash (NETZSCH, Germany). The thermal diffusivity was calculated using Eq. (1) ^40,41^:

1 $${\rm{C}}={{\rm{\alpha }}{\rm{t}}}_{50}{/{\rm{d}}}^{2},$$

where C is a dimensionless constant, α is the thermal diffusivity, t_50_ is the time required to achieve half of the maximum temperature on the rear, and d denotes the sample thickness. The thermal conductivity was calculated based on Eq. (2):

2 $${\rm{k}}=({\rm{\alpha }})\,({\rm{\rho }})\,({\rm{Cp}}),$$

where k, ρ, and Cp represent the thermal conductivity, bulk density, and specific heat of the samples, respectively. The density (ρ) was calculated by the conventional method using the equation: ρ = m/V = m/abc, where m, a, b, c are the mass, length, width, and thickness of the samples. For each kind of composites, five samples were prepared and four measurements were repeatedly tested for every sample material, and then an average value was calculated and utilized. The uncertainty and error of replicate measurements for each single material sample are tried to be minimized to get the figures of merit of thermal conductivity. As five samples were determined for every composite sample, the material productions are reproducible.
